# Economic evaluation of a guided and unguided internet-based CBT intervention for major depression: Results from a multi-center, three-armed randomized controlled trial conducted in primary care

**DOI:** 10.1371/journal.pone.0172741

**Published:** 2017-02-27

**Authors:** Pablo Romero-Sanchiz, Raquel Nogueira-Arjona, Antonio García-Ruiz, Juan V. Luciano, Javier García Campayo, Margalida Gili, Cristina Botella, Rosa Baños, Adoración Castro, Yolanda López-Del-Hoyo, Mª Ángeles Pérez Ara, Marta Modrego-Alarcón, Fermín Mayoral Cleríes

**Affiliations:** 1 Mental Health Clinical Management Unit, Institute of Biomedical Research of Malaga (IBIMA), Regional University Hospital Carlos Haya, Malaga, Spain; 2 Department of Personality, Assessment and Psychological Treatments, Faculty of Psychology, University of Malaga, Malaga, Spain; 3 Chair of Health Economics and Rational Drug Use. Department of Pharmacology and Pediatrics, Faculty of Medicine, University of Malaga, Malaga, Spain; 4 Teaching, Research & Innovation Unit, Parc Sanitari Sant Joan de Déu, St. Boi de Llobregat, Spain; 5 Primary Care Prevention and Health Promotion Research Network, RedIAPP, Madrid, Spain; 6 Aragon Institute for Health Research (IIS Aragon), Zaragoza, Spain; 7 University Hospital Miguel Servet, Zaragoza, Spain; 8 IUNICS-IDISPA, University of Balearic Islands, Palma de Mallorca, Spain; 9 University Jaume I, Castellon de la Plana, Spain; 10 CIBER Physiopathology Obesity and Nutrition (CB06/03) Carlos III Health Institute, Madrid, Spain; 11 University of Valencia, Valencia, Spain; 12 University of Zaragoza, Zaragoza, Spain; 13 Foundation Institute of Health Research of Aragon (IIS Aragon), Zaragoza, Spain; TNO, NETHERLANDS

## Abstract

Depression is one of the most common mental disorders and will become one of the leading causes of disability in the world. Internet-based CBT programs for depression have been classified as “well established” following the American Psychological Association criteria for empirically supported treatments. The aim of this study is to analyze the cost effectiveness at 12-month follow-up of the Internet-based CBT program “Smiling is fun” with (LITG) and without psychotherapist support (TSG) compared to usual care. The perspective used in our analysis is societal. A sample of 296 depressed patients (mean age of 43.04 years; 76% female; BDI-II mean score = 22.37) from primary care services in four Spanish regions were randomized in the RCT. The complete case and intention-to-treat (ITT) perspectives were used for the analyses. The results demonstrated that both Internet-based CBT interventions exhibited cost utility and cost effectiveness compared with a control group. The complete case analyses revealed an incremental cost-effectiveness ratio (ICER) of €-169.50 and an incremental cost-utility ratio (ICUR) of €-11389.66 for the TSG group and an ICER of €-104.63 and an ICUR of €-6380.86 for the LITG group. The ITT analyses found an ICER of €-98.37 and an ICUR of €-5160.40 for the TSG group and an ICER of €-9.91 and an ICUR of €496.72 for the LITG group. In summary, the results of this study indicate that the two Internet-based CBT interventions are appropriate from both economic and clinical perspectives for depressed patients in the Spanish primary care system. These interventions not only help patients to improve clinically but also generate societal savings.

Trial Registration: clinicaltrials.gov NCT01611818

## Introduction

Depression is the most common single mental disorder and will be one of the leading causes of disability worldwide in the coming years [1]. In the Spanish general population, the 12-month prevalence is estimated at 3.96% (2.15% in men and 5.62% in women), and the lifetime prevalence is estimated at 10.55% (6.29% in men and 14.47% in women) [2]. The incidence of depression across all ages, the trend toward chronicity and the risk of suicide make depression one of the most devastating diseases in terms of societal costs. In 2004, the total annual cost of depression in Europe was estimated to be €118 billion, or €253 per inhabitant [3]. The societal cost of affective disorders in Spain was estimated in 2010 to be €10,763 million, or €3584 per patient/year [4]. The vast majority of these patients are diagnosed and treated by their general practitioners [5]. Recent studies indicate that 10% to 16% of primary care patients fulfill the criteria for a diagnosis of major depression [6,7].

Pharmacological treatment is the standard for the management of depressed patients in primary care, including those who suffer minor or less severe symptoms. Approximately 70% of patients with a mood disorder in Spanish primary care receive psychotropic drugs [8]. A recent meta-analysis showed that the effect of antidepressants is non-existent or negligible among depressed patients with mild or moderate symptoms [9]. The recommendations of clinical guidelines for depression advise the active monitoring of symptoms [10] or collaborative strategies with specialized services and a stepped model of care [11] in which psychological treatments play a crucial role. In general, psychotherapeutic interventions aim to help patients identify how past and present factors may contribute to their depression and to teach them to deal effectively with these factors. Various studies suggest that patients prefer psychological treatments over drugs [12,13].

Cognitive-behavioral therapy (CBT) has received strong support in the treatment of depression [14]. A recent systematic review based on 11 randomized controlled trials (RCTs) with 1511 patients suggests no difference in the treatment effects of second-generation antidepressants and CBT, either alone or in combination [15]. Nevertheless, psychological therapies remain difficult to access, especially in primary care, due to the lack of highly trained and qualified practitioners [16]. Computerized psychological therapies offer an alternative to overcome accessibility barriers and to reduce the stigma associated with treatments in mental health settings. Internet interventions have demonstrated effectiveness for depression [17]. Moreover, online CBT programs for depression, social phobia and panic disorder have been classified as “well established” following the American Psychological Association criteria for empirically supported treatments [18,19]. Cost-effectiveness evaluations are still scant, but the available evidence suggests that online CBT treatments are cost effective compared with no treatment or face-to-face CBT [20,21].

Recently, an RCT was conducted in Spain to evaluate an Internet-delivered, CBT-based self-help program called “Smiling is fun”, provided with or without psychotherapist support, compared with usual care for patients with mild and moderate depression in primary care settings. The results of this study revealed a significant reduction of depressive symptoms at 3-month and 12-month follow-ups [22]. The effect size of the primary clinical measure at the 12-month follow-up was d = .73 for “improved treatment as usual” (iTAU) (medium effect), 1.2 for “totally self-guided” (TSG) (very large effect), and 1.22 for “low intensity therapist-guided” (LITG) (very large effect). At the 12-month follow-up, 72.4% and 75% of the TSG and LITG patients, respectively, had accessed the program, and 41.8% and 50%, respectively, had completed >6 modules. Only 13 participants (13.5%) in the LITG group contacted a therapist in the program via email.

The aim of this study is to investigate the cost effectiveness and cost utility of the Internet-based CBT program “Smiling is fun” with and without psychotherapist support compared to usual care at 12-month follow-up from a societal perspective, which means that both direct and indirect costs are analyzed.

## Methods

### Design

This study comprises an economic evaluation and a 12-month, pragmatic, multi-center, three-armed parallel RCT. The perspective used in our analysis is societal. Given that the time horizon is 12 months, it is not necessary to apply a discount factor to the costs.

### Setting, patients and randomization

The RCT was conducted in 30 Spanish primary care centers in four Spanish regions (Aragon, Andalusia, Baleares and Valencia) from November 2012 to June 2015. It is important to clarify that Spanish health care is publicly financed with universal coverage. Care delivery is arranged in health catchment areas with populations ranging from 5,000 to 25,000, which cover the entire territory.

Adults presenting depressive symptoms in primary care were randomized to receive either improved treatment as usual from their general practitioner (GP) or an Internet-based CBT intervention program (“Smiling is Fun”) for depression with or without psychotherapist support. The protocol of the study [23], the manual used to implement the program [24], and a study of the expectations of depressed primary care patients regarding this type of intervention have been published elsewhere [25,26].

For the clinical evaluation, we conducted assessments at baseline, post-treatment (3 months post-baseline assessment) and 3-month and 12-month follow-ups. For the present work, only data collected at the baseline (between November 2012 and January 2014) and at the 12-month follow-up (between March 2013 and June 2015) were used.

**Fig 1:**
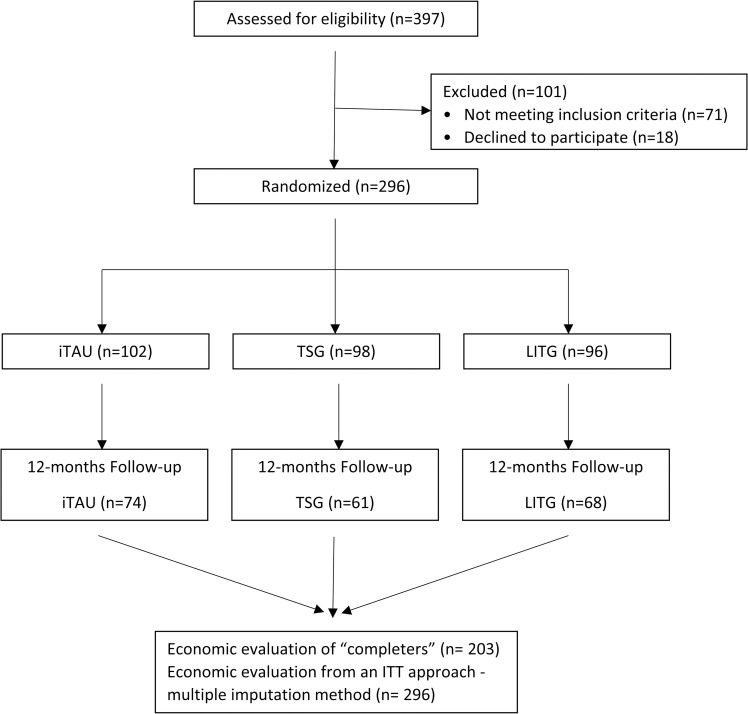
CONSORT flowchart of the participants in the economic evaluation. iTAU: improved treatment as usual; TSG: totally self-guided Internet-based program; LITG: low-intensity therapist-guided Internet-based program.

A total of 296 patients were recruited for the study. The recruitment stopped after the estimated sampled size was reached. Fig 1 presents a flowchart of the process of sampling, randomizing, and evaluating the patients, from the selection of the patients by the GPs to the 12-month follow-up. An independent statistician randomly allocated 102 patients to the iTAU group, 98 patients to the TSG group and 96 patients to the LITG group. The randomization was implemented using a computer-generated random number sequence by an independent statistician employing blocked randomization. The nature of the interventions made it virtually impossible to keep the participants blind to the assignments. However, the outcome evaluators were blind to the participants’ allocation.

The inclusion criteria of the study were a) aged 18–65 years; b) able to understand and read Spanish; c) mild or moderate severity symptoms according to the Spanish version of the Beck Depression Inventory-II (BDI-II) [27] (14–19: mild depression; 20–28: moderate depression); d) symptoms lasting longer than 2 weeks; e) access to Internet at home; and f) an email account. The exclusion criteria were a) attendance at any psychological treatment during the last year and b) severe psychiatric disorder in Axis I (alcohol/substances abuse or dependence, psychotic disorders, bipolar disorders, or dementia). Patients who were taking antidepressant medication were allowed to participate in the study, provided that the medication was prescribed at least four weeks before the patients’ inclusion in the study. However, patients whose dosage of antidepressant medication was increased during the study were considered drop-outs.

GPs referred the patients to psychologists, who interviewed the patients to assess the inclusion and exclusion criteria. Major depression symptoms were identified using the Mini-International Neuropsychiatric Interview [28,29]. After providing signed informed consent, a baseline battery of measures was administered to the participants by blind evaluators.

The study was approved by the local research and ethics committees (the Regional Ethics Committee of Aragón, the Regional Ethics and Research Committee of Málaga, the Deontological Commission of University Jaume I, and the Research Ethics Committee of Illes Balears). All the participants provided signed informed consent. This study was conducted according to the principles expressed in the Declaration of Helsinki. A detailed description of the RCT protocol and the effectiveness results can be accessed elsewhere [22,23].

### Interventions

#### Control intervention

All the patients included in the study (whether in the control or intervention arms) received usual care from their GPs. This treatment as usual was provided by GPs who participated in a three-hour training program to update their knowledge of how to treat depression in primary care based on the NICE recommendation [11]. The program included the use of antidepressants in adequate doses and lengths of time. The protocol indicated that if suicide risk, severe social dysfunction, or worsening of symptoms were detected, patients should be referred to the appropriate mental health care unit.

#### Internet-based CBT interventions

“Smiling is fun” is an Internet-delivered self-help program for the treatment of depression based on similar programs that have demonstrated effectiveness in other countries [17,30,31]. The program consists of ten CBT modules covering different psychological techniques to cope with depression. These modules are sequential; thus, each module is followed by a pre-designated module. The program recommends working on a single module for at least one week. The modules are as follows: 1. medication management; 2. sleep hygiene; 3. motivation for change; 4. understanding emotional problems; 5. learning to move on; 6. learning to be flexible; 7. learning to enjoy; 8. learning to live; 9. living and learning; and 10. from now on, what else? The content of the modules can be found elsewhere [23,24]. Two intervention groups were available: LITG and TSG. In the first, a trained psychotherapist contacted patients by email to offer help. The support included help to overcome difficulties in making progress with the program as well as help with technical questions. In the second group, help was offered only for technical questions.

### Study measures

#### Beck Depression Inventory-II (BDI-II) [32]

The primary clinical variable was the severity of depression, which was measured using the Spanish version of the BDI-II [33]. This inventory consists of 21 items with response options from 0–3. Therefore, the total range of the scale is from zero to 63. The BDI-II is one of the most widely used self-report measures of depression symptoms and includes cognitive, emotional and somatic symptoms. Several studies show that this instrument has good psychometric properties [32] and good agreement with the clinical diagnosis of depression [34]. The BDI-II scores were used as outcomes in the cost-effectiveness analysis.

#### EuroQol-5D-3 L (EQ-5D-3 L) [35]

The secondary clinical variable was health-related quality of life (HRQoL). The utility scores were obtained from the EuroQol-5D (EQ-5D) classification system and were used to rate the patients’ HRQoL on a scale from 0 (as bad as death) to 1 (perfect health). Negative values are possible and indicate a health state that is “worse than death”. These scores reflect how the general population values the health status described by the subject, which is preferred for economic evaluations from a broad perspective. In our case, quality-adjusted life years (QALYs) were calculated on the basis of these scores using the Spanish tariffs of the EQ-5D [35]. The EQ-5D is designed to assess generic quality of life and can be used with a broad range of patient groups, including patients with depression [36,37].

#### Client Service Receipt Inventory (CSRI) [38]

Data on the use and utilization of health and social services were collected using the Spanish version of the Client Service Receipt Inventory (CSRI) [38]. The variant used in this study was designed to retrospectively collect data on service use during the previous 12 months.

### Sample size

The estimated sample size for the protocol was 450 patients [23]. However, recruitment challenges in one of the target regions, which delayed recruitment more than one year, forced us to forego recruitment in that region. The sample size was recalculated to 300 patients using less conservative criteria. A detailed description of the new sample size calculation can be reviewed in the protocol published elsewhere [22].

### Statistical analyses

The data collection and statistical analyses were performed using SPSS (SPSS Inc., Chicago, IL, USA) software version 20, licensed for the University of Malaga.

Frequency and proportions were used for descriptive statistics of the categorical and qualitative variables. Means and standard deviations were calculated for the quantitative variables. Comparisons between groups were performed using chi-square tests for qualitative variables and one-way ANOVAs for quantitative variables. Size effects were calculated using Cohen’s d. In all cases, statistical significance corresponded to a value of p<0.05.

The calculation of costs, effectiveness and utility were performed using two perspectives. First, we conducted a complete case analysis with the 203 patients who completed the 12-month follow-up. Second, the analyses were repeated using an intention-to-treat (ITT) approach (sensitivity analysis) with the 296 randomized patients. The imputation of missing data was performed using the iterative Markov chain Monte Carlo (MCMC) technique. It was assumed that data were missing at random.

#### Description of cost procedure

Costs were estimated from the healthcare and societal perspectives during the 12 months before the baseline and during the 12 months of follow-up. Direct health care costs were calculated by adding the costs derived from medication consumption, medical tests, and the use of health-related services (see Table 1).

**Table 1:** Unit costs used in the calculation of direct and indirect costs (financial year 2014; values in €).

Type of utilization		Unit cost (€)
Cost in the healthcare system	General practitioner (per appointment)	27.5
	Nurse or psychiatric nurse (per appointment)	25.8
	Social worker (per appointment)	24.4
	Psychologist (per appointment)	43.9
	Psychiatrist (per appointment)	43.9
	Other medical specialists (per appointment)	65.2
	Hospital emergency visits (per attendance)	165.9
	Hospital stays (per night)	641.7
	Diagnostic tests (range)	4.3 to 375.8
Productivity losses	Absenteeism from work (minimum daily wage)	21.5

The cost of medication was calculated by determining the price per milligram according to the databases of the drug from the Ministry of Health and Consumer and included the value-added tax. The total costs of medications were calculated by multiplying the price per milligram by the daily dosage used (in milligrams) and the number of days that the treatment was received. The main source of the unit cost data for medical tests and health services was the OBLIKUE database of health care costs (available at Oblikue Consulting. Base de Datos de Costes Sanitarios eSALUD Barcelona; 2014. http://www.oblikue.com/bddcostes). The OBLIKUE database contains information about Spanish healthcare service costs and is derived by systematic reviews of the literature. It consists of approximately 18,000 entries. Table 1 shows the unit costs of healthcare resources. Indirect costs (lost productivity) were calculated using the human capital approach, which involved multiplying the minimum daily wage in Spain for 2014 by the number of days of sick leave as reported by each patient. Finally, total costs were calculated by adding the direct and indirect costs. Unit costs are expressed in Euros (€) based on 2014 prices.

The three interventions in this study were conducted by public agencies. The iTAU intervention was conducted by the Spanish National Health Service as part of its training program. The development of the Internet-based CBT program was funded by the Carlos III Institute of Health; its utilization by the Spanish National Health Service will not involve further costs. Therefore, no costs associated with the interventions will be included in the study.

The economic evaluation of this study follows the general guidelines for conducting pharmacoeconomic analyses in Spain. It also follows the Consolidated Health Economic Evaluation Reporting Standards (CHEERS) [39] and the Guidelines of the International Society for Pharmacoeconomics and Outcomes Research (ISPOR) [40].

#### Cost-effectiveness and cost-utility analysis

The effectiveness of the interventions was estimated using the difference between the BDI-II score at the baseline and at the 12-month follow-up, and the utility was estimated using QALYs at the 12-month follow-up.

Cost effectiveness was explored through the calculation of incremental cost-effectiveness ratios (ICERs) for the active intervention groups (TSG and LITG) using the iTAU group as the control. ICER is defined as the ratio between incremental costs and incremental effectiveness. ICERs were estimated using the following formula:

$$ICER=\frac{Cost\;active\;intervention\;group-\;Cost\;control\;intervention\;group}{Efectiveness\;or\;Utility\;active\;intervention\;group-\;Efectiveness\;or\;Utility\;control\;intervention\;group}.$$

In the same way, cost utility was explored through the calculation of incremental cost-utility ratios (ICURs), defined as the ratio between incremental costs and incremental utilities measured on QALYs. QALYs were approximated using the area-under-the-curve technique. ICURs were calculated using the same equation but employing QALYs instead of clinical units (BDI-II points).

Two cost-utility planes were plotted, one for each contrast (TSG vs. iTAU and LITG vs. iTAU). In a cost-utility plane, the incremental costs between an intervention group and a control or usual care group are plotted on the y-axis, and the incremental utility is plotted on the x-axis. The resulting four quadrants represent the following: the northeast quadrant indicates that the intervention is more useful and more expensive than the control intervention; the southeast quadrant indicates that the intervention is more useful and less expensive and is said to dominate the control intervention; in the southwest quadrant, the active intervention is less useful and less expensive; and in the northwest quadrant, the intervention is less useful and more expensive and is said to be dominated.

## Results

### Attrition rate

The analyses showed that the patients from the three groups were comparable in terms of sociodemographic and clinical features (see Table 2). Most of the patients were women with families and university-level education who were employed. Most of the patients used some type of medication and had a mean score on the BDI-II of 23 (moderate depression).

**Table 2:** Baseline sociodemographic characteristics of the patients by treatment group.

Characteristics at baseline	iTAU (n = 102)	TSG (n = 98)	LITG (n = 96)
**Age,** *Mean (SD)*	43.04 (9.66)	42.57 (11.94)	43.19 (9.30)
**Sex** *(%)*			
female	76 (74.5)	72 (73.5)	76 (79.2)
**Living** *(%)*			
with family	92 (90.2)	90 (91.8)	82 (85.4)
**Level of studies** *(%)*			
university	30 (29.4)	29 (29.6)	32 (33.3)
**Employment** *(%)*			
employed	54 (34.4)	51 (32.5)	52 (33.1)
**Income** *(%)*			
<1 NMW	27 (26.5)	34 (34.7)	22 (22.9)
1–2 NMW	42 (41.2)	33 (33.7)	40 (41.7)
≥3 NMW	33 (32.4)	31 (31.6)	34 (35.4)
**Antidepressant medication,** *(%)*			
yes	91 (89.2)	84 (85.7)	88 (91.7)
**Physician visits,** *Median (Q*_*1*_*-Q*_*3*_*)*	5 (2–8)	5 (3–10)	5 (3–8)

iTAU: improved treatment as usual; TSG: totally self-guided Internet-based program; LITG: low-intensity therapist-guided Internet-based program. NMW: national minimum wage.

Data for the calculation of the costs of the clinical resources used by the patients in the last year were obtained at the baseline assessment and 12 months post-treatment. One hundred percent of the patients completed the assessment at the baseline, and 68.6% completed it at the 12-month follow-up. No statistically significant differences between the three groups (iTAU = 27.5%, TSG = 37.8%, LITG = 29.2%) were found for attrition rate (χ^2^ = 0.415; P = .812). Fig 1 illustrates the flow of participants through the economic evaluation.

### Baseline and 12-month follow-up costs

Table 3 shows the disaggregated direct and indirect costs by each group of patients at the baseline and 12-month follow-up assessments. One-way ANOVA analyses found that the three groups were comparable in terms of indirect (p = 0.129), direct (p = 0.668) and total costs (p = 0.537) during the year before the baseline assessment. However, statistically significant differences were found in the 12-month follow-up analyses of the completers for indirect (p = 0.001) and total costs (p = 0.005) but not for direct costs (p = 0.263). Indirect costs were significantly higher in the LITG group, which produced higher costs for sick leaves and productivity loss. The TSG group’s total net costs were significantly lower than those of the other two groups. This difference was due to the higher indirect costs of the LITG group and the higher direct costs of the iTAU group. The total net costs of the TSG group patients were €700–800 lower than the other two groups. In the analyses using an ITT perspective, no statistically significant differences were found between the costs of the three groups.

**Table 3:** Costs (total and disaggregated) and outcomes by treatment groups and study period.

Baseline (n = 296)	1. iTAU (n = 102)	2. TSG (n = 98)	3. LITG (N = 96)	F or χ^2^	p value
Costs (€)					
Direct medical	1,455.06 (1,473.65)	1,892.08 (2,348.07)	1,397.65 (1,667.21)	2.062	0.129
Medication	67.27	111.70	88.84		
Indirect costs	1,370.06 (2,346.68)	1,115.16 (1,891.88)	1,140.67 (2,366.97))	0.404	0.668
Sick leaves	378.99	448.40	331.22		
Productivity loss	991.06	666.76	809.45		
Net total costs	2,892.39 (2,889.71)	3,118.94 (3,125.60)	2,627.17 (3,198.88)	0.623	0.537
Outcomes					
Effectiveness (BDI-II score)	22.27 (5.52)	22.44 (4.94)	22.40 (5.29)	0.029	0.952
Utility (EQ-5D Utility score)	.7076 (.1482)	.6989 (.1417)	.6794 (.1785)	0.834	0.435
Completers 12-month follow-up (n = 203)	iTAU (n = 74)	TSG (n = 61)	LITG (N = 68)	F or χ^2^	p value
Costs (€)					
Direct medical	1562.49 (2913.2)	918.38 (1848.15)	1083.29 (2145.8)	1.366	0.258
Medication	51.06	62.79	64.32		
Indirect costs	508.88 (641.97)	421.64 (1173.39)	569.54 (450.13)	0.560	0.572
Sick leaves	301.33	338.62	349.02		
Productivity loss	207.55	83.02	220.52		
Net total costs	2122.43 (775.04)	1402.81 (429.64)	1717.15 (509.49)	24.345	0.000
Outcomes					
Effectiveness (BDI-II score)	15.94 (11.06)	12.32 (10.94)	11.59 (11.48)	3.093	0.048
Utility (EQ-5D Utility score)	.7059 (.2221)	.7626 (.2401)	.7810 (.2050)	2.213	0.112
ITT 12-month follow-up (n = 296)	iTAU (n = 102)	TSG (n = 98)	LITG (N = 96)	F or χ^2^	p value
Costs (€)					
Direct medical	1,156.35 (1,742.21)	822.70 (1,397.62)	848.42 (1,442.96)	1.461	0.234
Medication	51.06	62.79	64.32		
Indirect costs	508.88 (1,375.40)	421.59 (1,474.52)	844.48 (2,852.59)	1.205	0.301
Sick leaves	262.41	294.46	250.36		
Productivity loss	246.47	127.12	594.12		
Net total costs	1,716.29 (2,436.93)	1,307.07 (2,218.66)	1,757.22 (3,636.51)	0.763	0.467
Outcomes					
Effectiveness (BDI-II score)	15.94 (10.99)	11.96 (11.92)	12.04 (10.79)	4.107	0.017
Utility (EQ-5D Utility score)	0.7059 (0.2209)	0.7852 (0,2427)	0.7883 (0.2029)	4.402	0.013

iTAU: improved treatment as usual; TSG: totally self-guided Internet-based program; LITG: low-intensity therapist-guided Internet-based program; ITT: intention-to-treat; BDI-II: Beck Depression Inventory II; EQ-5D: EuroQol-5D; ICER: incremental cost-effectiveness ratio; ICUR: incremental cost-utility ratio.

### Cost utility and cost effectiveness

Both the utility and effectiveness measure scores were comparable among the three groups at the baseline (see Table 4). However, statistically significant differences were found at the 12-month follow-up for effectiveness but not for the utility measures in the completers’ analyses. Both active interventions (TSG and LITG) were more effective than the iTAU intervention. Again, the ITT analyses showed that both Internet-based CBT interventions were more effective and useful than iTAU.

**Table 4:** Incremental cost, effect, utility, and cost-effectiveness/cost-utility ratios from the societal perspective.

	Incremental cost	Incremental effectiveness (reduction in BDI-II)	Incremental utility (QALY)	ICER	ICUR
Completers					
TSG (vs. iTAU)	-644.11	3.80	0.0567	-169.50	-11389.96
LITG (vs. iTAU)	-479.20	4.58	0.0751	-104.63	-6380.86
ITT					
TSG (vs. iTAU)	-409.22	4.16	0.0793	-98.37	- 5,160.40
LITG (vs. iTAU)	40.93	4.13	0.0824	9.91	496.72

iTAU: improved treatment as usual; TSG: totally self-guided Internet-based program; LITG: low-intensity therapist-guided Internet-based program; ITT: intention-to-treat; BDI-II: Beck Depression Inventory II; QALY: quality-adjusted life year; ICER: incremental cost-effectiveness ratio; ICUR: incremental cost-utility ratio.

The analyses of the completers’ sample showed that the ICER of TSG compared with iTAU was €-169.50, which indicates that each point of improvement in the BDI-II using TSG instead of iTAU was accompanied by savings of €169.50. The ICER of LITG compared with iTAU was €-104.63, which indicates that each point of improvement in the BDI-II using that intervention was also accompanied by savings of €104.63. The analyses using the ITT perspective showed that the ICER of TSG compared with iTAU was €-98.37, which also indicates that each point of improvement using TSG saved €98.37. Finally, the ICER of LITG compared with iTAU was €9.91, which indicates that each point of improvement in the BDI-II using that intervention involved an extra cost of €9.91.

Regarding cost utility, using the completers’ sample, the ICUR of TSG compared with iTAU was €-11389.96, which indicates that each extra QALY using the TSG intervention instead of iTAU also saved €11389.96. The ICUR of LITG compared with iTAU was €-6380.86; that is, the LITG intervention saved €-6380.86 for each QALY gained by this intervention. The analyses using the ITT perspective found that the TSG intervention saved €-5160.40 and that the LITG cost €496.72 for each extra QALY gained using those interventions instead of iTAU.

**Fig 2:**
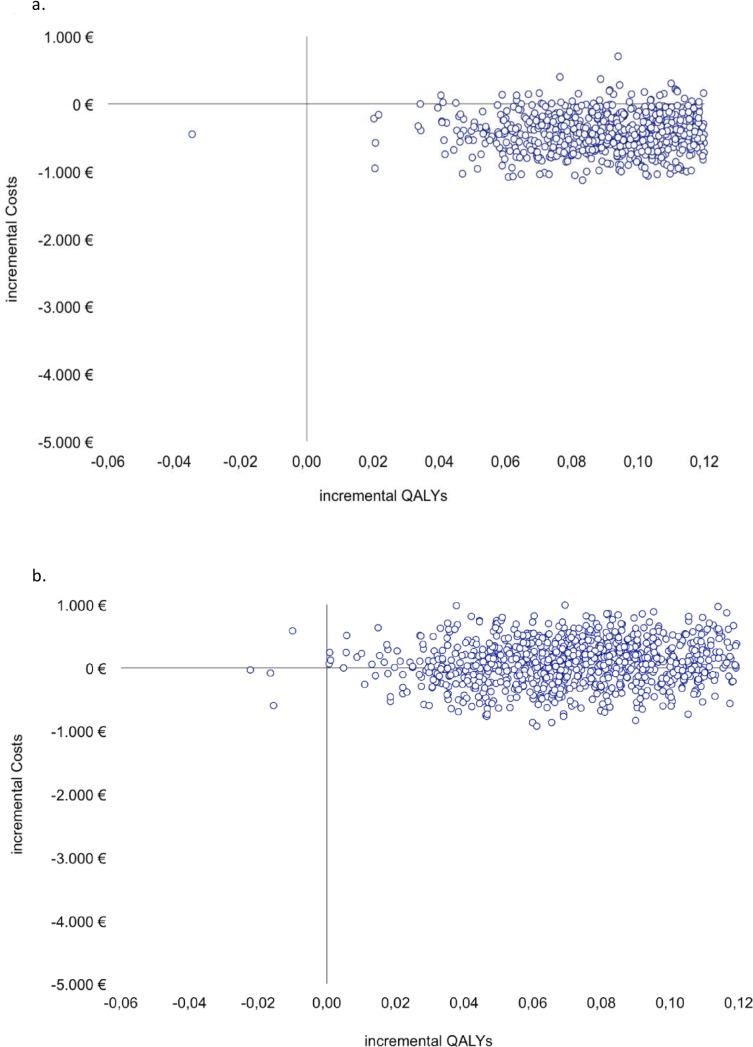
Cost-utility planes. Fig 2a. Scatterplot TSG vs. iTAU. Fig 2b. Scatterplot LITG vs. iTAU. QALY: quality-adjusted life year.

The cost-utility scatterplots (Fig 2A & 2B) graphically illustrate that both active interventions exhibited more utility than the control intervention given that most of the cost-utility pairs were located in the east quadrants. The TSG vs. iTAU scatterplot (Fig 2A) shows that this intervention is likely to be more effective and even less expensive than iTAU (southeast quadrant). However, the LITG vs. iTAU scatterplot (Fig 2B) indicates that this intervention is likely to be more effective than iTAU but shows more uncertainty about the cost given that the cost-utility pairs are located along the x-axis.

## Discussion

The aim of this study was to investigate the cost utility and cost effectiveness compared to usual care of two Internet-based CBT interventions for moderate and mild depression in primary care from a societal perspective. Although this type of intervention has been implemented in several countries and for many physical and mental problems, this is the first study to evaluate the cost effectiveness and cost utility of Internet-based CBT for Spanish patients with mild/moderate depression recruited in primary care.

The results of this study showed that both the TSG and LITG interventions were more effective and exhibited more utility than the iTAU intervention. Additionally, the ICER values indicated that each patient treated with either of the Internet-based CBT interventions resulted in cost savings compared to the iTAU intervention using both the complete case and ITT perspectives. The cost-utility analyses found that both Internet-based CBT interventions were superior to the iTAU intervention. Moreover, using the complete case analysis perspective, both interventions exhibited more utility (as described above) and even saved costs compared to iTAU. According to the ITT perspective, the LITG intervention produced slightly higher costs than iTAU but exhibited more utility. However, those costs were far below the threshold proposed by the National Institute for Clinical Excellence (£20,000) and the Spanish Ministry of Health (from €21,000 to €25,000) [41,42].

In summary, the results of this study indicate that both Internet-based CBT interventions showed cost utility and cost effectiveness compared with a control group. However, the decision regarding which treatment should be implemented in a health service is complex, even though the interventions used in this study not only displayed more utility and effectiveness but also saved costs compared with iTAU. From a purely clinical perspective, the LITG intervention exhibited more efficacy and utility, but TSG showed clinical results that were nearly as good as LITG and saved costs. Moreover, non-reported data revealed that both interventions reduced the number of referrals to mental health professionals during the 12-month follow-up period. Eighteen patients from the iTAU group, 9 from the TSG group and 8 from the LITG group were referred to a psychiatrist, and 13 patients from the iTAU group, 5 from the TSG group and 2 from the LITG group were attended by a psychologist during that period. This reduction could have led to reductions in the cost of both the TSG and LITG interventions compared with iTAU.

Compared with previous studies, the results of this study are quite positive. A systematic review by Donker and collaborators [43] claims that the clinical effect of this type of intervention on depression is strong but that its cost effectiveness is weak. The discrepancy in the results might be due to several reasons. First, the time horizon of our study (12 months) compared to other studies that reported ICERs should account for some variance, from 6 weeks [44] to 8 months [45]. In fact, a study with a broader time horizon showed similar results, indicating that Internet-based CBT interventions saved costs in comparison to control conditions from cost-effectiveness and cost-utility perspectives. Second, the indices used for effectiveness should also account for some variation. In our study, the difference in the main clinical measure was used, but in other studies, indices such as differences in the percentage of patients recovered or the number needed to treat were used. Third, the provenance of the patients and their level of depression may also account for some differences. Finally, cross-cultural variations in the management of depression in health services could explain the differences between studies.

This study and the conclusions drawn from its results are limited in some respects. First, although the attrition rate was good, it might limit the results. However, imputation of missing values confirmed the results [22]. Second, although the sample sizes provided sufficient statistical power, larger samples should allow the exploration of differences in costs, cost effectiveness and cost utility in different subsamples, such as people of different ages or men versus women.

In summary, the results of this study indicate that Internet-based CBT interventions have both cost effectiveness and cost utility for depressed patients in the Spanish primary care system. This type of intervention not only helps patients to improve clinically but also generates societal savings.

## Supporting information

S1 CONSORT Checklist (PDF)

S1 Protocol Low Intensity Versus Self-guided Internet-delivered Psychotherapy for Major Depression: A Multicenter, Controlled, Randomized Study. (PDF)
