# Supplementary material for: Economic evaluation of a guided and unguided internet-based CBT intervention for major depression: Results from a multi-center, three-armed randomized controlled trial conducted in primary care
Source: PLoS One. 2017 Feb 27;12(2):e0172741. doi: 10.1371/journal.pone.0172741 (PMC5328395; doi:10.1371/journal.pone.0172741)
Supplement: S1 Protocol — (PDF) [file pone.0172741.s002.pdf]

**CLINICAL PROTOCOL**

# Low Intensity Versus Self-guided Internet-delivered Psychotherapy for Major Depression: A Multicenter, Controlled, Randomized Study

**Status: Approved**

**Trial Registration:** NCT01611818

**Prepared by:** Fermín Mayoral Cleríes and Pablo Romero Sanchiz.

Regional University Hospital of Malaga.

## TABLE OF CONTENTS


1. BACKGROUND ..... 3
2. OBJECTIVES AND HYPOTHESES ..... 3
Objectives: ..... 3
Hypotheses: ..... 3
3. STUDY DESIGN ..... 4
Phases: ..... 4
Design: ..... 5
4. STUDY POPULATION ..... 5
Inclusion criteria ..... 5
Exclusion criteria ..... 6
5. TREATMENT ALLOCATION AND BLINDING ..... 6
Procedure and randomization ..... 6
6. INTERVENTION PROCEDURE ..... 7
Control intervention ..... 7
Computerized CBT interventions ..... 7
7. EVALUATIONS ..... 7
Measures: ..... 7
Schedule: ..... 8
8. STATISTICAL METHODS ..... 9
Sample size ..... 9
10. ETHICAL ASPECTS ..... 9
11. REFERENCES ..... 9

## 1. BACKGROUND

Major depression will become the second most important cause of disability in 2020 according to World Health Organization reports [1]. Computerized cognitive-behavior therapy could be an efficacious and cost-effective option for its treatment, particularly in patients with mild or moderate depression. In fact, several studies support the efficacy of these type of interventions in patients with mild and moderate depression [2]. The expansion of the interventions based on computerized cognitive-behavior therapy has been motivated, among other reasons, in its cost-effectiveness [3]. However, no studies on cost-effectiveness of low intensity vs self-guided psychotherapy have been carried out in a Spanish sample.

## 2. OBJECTIVES AND HYPOTHESES

**Objectives:**

1) To develop a computerized cognitive-behavior therapy program for the treatment of depression adapted to Spanish culture, language and population.
2) To evaluate the effectiveness of a computer-based psychotherapy program in Spanish for the treatment of depression in primary care.
3) To evaluate the cost-effectiveness and cost-utility of the computer-based psychotherapy program in Spanish for the treatment of depression

**Hypotheses:**

1) It is feasible to develop a computerized cognitive-behavior therapy program for the treatment of depression adapted to Spanish culture, language and population.

2) Computerized cognitive-behavior therapy for mild and moderate depression in primary care is effective in Spanish population.

3) Computerized cognitive-behavior therapy for mild and moderate depression in primary care is cost-effective in Spanish population.

**3. STUDY DESIGN:**

**Phases:**

Phase 1: Development of a computer-based psychotherapy program in Spanish for the treatment of depression:

1) Analysis of the programs for the treatment of depression developed in other contexts, such as “Beating the blues”, “Blues Begone”, “Living Life to the Full” or “Moodgym welcome”.

2) Analysis of the self-help/bibliotherapy programs based on CBT interventions for the treatment of depression developed in Spanish.

3) Development of an interactive computer-based program for the treatment of depression equivalent to 10 sessions of face-to-face CBT.

4) Development of a restricted-access web page which host the program.

5) Pilot study with N=20 patients on each centerto assess the comprehensibility, feasibility and usability of the program.

Phase 2: Randomized-controlled trial of an Internet-based intervention for depression in primary care in Spain

**Design:**

Randomized controlled trial, three-armed, with parallel assignment, double-blind study.

| **Arms** | **AssignedInterventions** |
| --- | --- |
| Low intensity Internet-delivered psychotherapy + improved treatment as usual by GP. | Low Intensity Internet-delivered psychotherapy<br>Patients will be contacted by a researcher trained in psychotherapy. Patients can ask for questions or advice to psychotherapists during the study |
| Self-guided Internet-delivered psychotherapy + improved treatment as usual | Self-guided Internet delivered psychotherapy<br>No contact with the therapists over the treatment period will be done. |
| Improved treatment as usual by GP | Improved treatment as usual<br>Any kind of treatment administered by the GP to the patient with depression |

**4. STUDY POPULATION**

**Inclusion criteria**

- Diagnosis of major depression. It will be carried out with MINI International Neuropsychiatric Interview + scoring of moderate or mild depression using Beck Depression Inventory II. Cut-off point for this questionnaire is: 0-13: minimal depression; 14-19: mild depression; 20-28: moderate depression; 29-63: severe depression [34, 35].
- Aged 18-65 years
- Able to understand and read Spanish language
- Moderate or mild major depression
- Duration of symptoms longer than 2 weeks
- Access to Internet at home and having an email address.

**Exclusion criteria**

- Any psychological structured treatment during last year
- Diagnostic of Severe psychiatric disorder in Axis I (alcohol/substances abuse or dependence, psychotic disorders or dementia) patients with severe depression (indicated by a Beck-II score of 29 or higher) who will be advised to consult their GP Receiving pharmacological treatment with antidepressants is not an exclusion criterion meanwhile, during the study period, treatment will not be modified or increased (decrease of pharmacological treatment is accepted).

## 5. TREATMENT ALLOCATION AND BLINDING

**Procedure and randomization**

The recruitment process will begin with the selection of the patients by the general practitioner (GP) as suitable for the study. The GP will give brief information about the program to each patient. If the patient is interested, the GP will ask for permission in order to collect personal information and to check the inclusion and exclusion criteria. The GP will inform about the candidate to the evaluator, who will set an appointment with the patient. In this appointment, the independent evaluator will give structured information to the patient about the study. If the patient agrees to participate, he/she will sign the informed consent form and the evaluator will assess the patient using the booklet of instrument. After the appointment, the evaluator will contact with an independent statistician who will assign the patient to one of the three arms of the study (randomization by blocks). After that, the statistician will get in touch with the patient to inform him about the assignment to one of the arms. After the baseline assessment, an independent evaluator, blind to the assignment, will assess the patients at the end of the treatment (three months after the baseline), and also at two follow-ups (3-months and 12-months).

## 6. INTERVENTION PROCEDURE

### Control intervention

All the patients of the study will receive improved usual care provided by their GPs. The general practitioners will receive a training course to improve their knowledge about mild and moderate depression.

### Computerized CBT interventions

Two separate computerized cognitive-behavior therapy interventions will be provided. In the first one (Self-guided Internet-delivered psychotherapy), access to de platform will be provided to the patients allocated in this arm. Only technical support will be provided to the patients who need it.

In the second intervention (Low intensity Internet-delivered psychotherapy), besides access to the platform, the patients will have the possibility to get in touch with a trained psychotherapist via email, and ask for individualized advice.

## 7. EVALUATIONS

### Measures:

*Primary Outcome Measures:*

- Severity of depressive symptomatology measured by Beck Depression Inventory II.

This is one of the most widely questionnaires used to evaluate severity of depression in pharmacological and psychotherapy trials. This questionnaire has been used because it is recommended to assess depression in primary care patients in which comorbidity with medical disorders is frequent. The Spanish validated version of the questionnaire will be used.

*Secondary Measures:*

- Socio-demographic variables. The following socio-demographic data will be collected: gender, age, marital status (single, married/relationship, separated/divorced, and widowed), education (years of education), occupation, economical level.

- EuroQoL-5D questionnaire (EQ-5D - Spanish version). Generic instrument of health-related quality of life. It has two parts: part 1 records self-reported problems in each of five domains: mobility, self-care, usual activities, pain/discomfort and anxiety/depression. Each domain is divided into three levels of severity corresponding to no problems, some problems, and extreme problems. Values range from 1 (best health state) to 0 (death). Part 2 records the subject's self-assessed health on a VAS, a 10 cm vertical line on which the best and worst imaginable health states score 100 and 0, respectively.

- Client Service Receipt Inventory - adapted (CSRI - Spanish version). Questionnaire for collecting information about use of healthcare and social care services and other economic impacts (such as time off work due to illness). The variant used in this study was designed to collect retrospective data on service utilization during the previous months after the last assessment. Data on baseline assess the previous three months before inclusion.

**Schedule:**

The patients will be assessed using the booklet by blinded evaluators at baseline, post-treatment (3 months after the first assessment), at 3-months follow-up and at 12-months follow-up.

The estimated time for the recruitment of the whole sample is one year, two years and a half including the 12-months follow-up.

## 8. STATISTICAL METHODS

The statistical analyses will follow the recommendations of the CONSORT statement. An intention-to-treat approach will be followed. First, a between group comparison of the main clinical and sociodemographic variables at baseline will be carried out in order to state the comparability of the groups. A mixed ANOVA 3x4 (three groups and four time points) with the main clinical measure (BDI-II) will be carried out in order to find group, time and mixed effects. Post-hoc analyses will be performed to detect specific differences between groups and time points. The same analyses will be performed with the secondary measures.

The economic evaluation will be estimated from a societal perspective during the 12 months before the baseline and during the 12-months of follow-up. Direct health care costs will be calculated by adding the costs derived from medication consumption, medical tests, use of health-related services, and cost of the staff running the CBT intervention.

### Sample size

Based on previous studies, a clinically significant difference in the main clinical measure used in this project (BDI-II) is 5 points. Assuming a statistical power of 90%, and apha of 0.05 and two tails, the sample size needed is 100 patients. Given that an attrition rate of 70% is expectable, 150 patients per group (three groups), 450 in total, is estimated an appropriate sample size.

## 9. ETHICAL ASPECTS

All patients included in this trial will be informed about every aspect related to ethical aspects of the study. Signed informed consent will be also required. Confidentiality will be granted following the Organic Law 15/1999. All procedures of this trial will follow the principles expressed in the Declaration of Helsinki.

## 10. REFERENCES

1. World Health Organization N. The Global Burden of Disease: 2004 update [Internet]. Update. 2008. doi:10.1038/npp.2011.85

2. Kaltenthaler E, Brazier J, De Nigris E, Tumur I, Ferriter M, Beverley C, et al. Computerised cognitive behaviour therapy for depression and anxiety update: a systematic review and economic evaluation. Health Technol Assess. England; 2006;10: iii, xi–xiv, 1-168. Available: http://eprints.whiterose.ac.uk/1774

3. McCrone P, Proudfoot J, Ryden C, Everitt B, Shapiro D a, Goldberg D, et al. Clinical efficacy of computerised cognitive-behavioural therapy for anxiety and depression in primary care: randomised controlled trial. Br J Psychiatry. 2004;185: 55–62. doi:10.1192/bjp.185.1.46
